# Executive functions and quality of life in children with neurofibromatosis type 1

**DOI:** 10.1186/s13023-021-02051-5

**Published:** 2021-10-09

**Authors:** Arnaud Roy, Jean-Luc Roulin, Christèle Gras-Le Guen, Marie-Laure Corbat, Sébastien Barbarot

**Affiliations:** 1 LPPL, SFR Confluences, Univ. Angers, Université de Nantes, 49000 Angers, France; 2 Reference Center for Learning Disabilities, Nantes University Hospital, Nantes, France; 3 Neurofibromatosis Clinic, Nantes University Hospital, Nantes, France; 4 Psychology and Neurocognition Laboratory (LPNC), CNRS-University of Grenoble-Alpes-University of Savoie Mont-Blanc, Grenoble, France; 5 Clinical Investigation Center, Nantes University Hospital, Nantes, France; 6 Physiology of Nutritional Adaptations (PhAN) Laboratory, INRA and Dermatology Department, Nantes University Hospital, Nantes, France; 7 Faculté des Lettres, Langues et Sciences Humaines, Université d’Angers, 11 Boulevard Lavoisier, 49045 Angers Cedex 01, France

**Keywords:** Executive dysfunction, Genetic disorder, Neuropsychology, Learning disabilities, Brain

## Abstract

**Background:**

To examine the impact of executive function disorders on health-related quality of life (QoL) in children with neurofibromatosis type 1 (NF1), we conducted a prospective single-center study among 40 children with NF1 aged 8–12 years (mean = 9.7, *SD* = 1.4) and their parents, comparing them with 56 healthy control children matched for age, sex, parental education level, and handedness. We collected children’s self-reports and parents’ proxy reports of QoL with the Kidscreen-52 questionnaire, and measured executive functions by combining seven performance-based tests and a daily life questionnaire completed by parents and teachers.

**Results:**

Several QoL domains were significantly impaired in the children with NF1, compared with healthy controls, mainly according to their parents’ reports (3 out of 9 scales; Cohen’s *d*: 0.57–0.76), with particularly low scores in the social support and peers and school environment domains. Executive function difficulties (Cohen’s *d*: 0.64–1.72) significantly predicted the impairment of QoL domains as perceived by the children or their parents, regardless of the indirect indicators of learning disabilities.

**Conclusions:**

Both performance-based executive function scores and behavioral ratings of executive functions in daily life by parents and teachers were associated with low QoL levels in the children with NF1. The school environment and social integration appear to be particularly affected and should therefore be targeted in the management of the disease.

## Background

Neurofibromatosis type 1 (NF1) is one of the most common autosomal dominant genetic diseases (1:3500 [1]). Children with NF1 have impaired quality of life (QoL) [2, 3]. Neuropsychological and learning disorders are frequent complications in these children (30–65% [4]), especially when they involve executive functions (EFs) [5, 6]. The latter are a set of high-level controlled processes, such as planning, inhibition, flexibility and working memory, that subtend appropriate goal-directed behaviors [7, 8]. It is now widely agreed that EFs are crucial for psychological development, academic success, and QoL [9].

Significant relationships between EF difficulties and poor QoL have been identified in various chronic pediatric disorders, including epilepsy [10], developmental coordination disorder [11], and autism spectrum disorder [12]. Although some sociodemographic or disease-related factors (e.g., familial vs. sporadic disease, disease severity) are thought to predict poor QoL in children with NF1 [3], the potential impact of neuropsychological disorders on QoL remains largely unknown.

In this context, the objective of the current prospective study was to examine the extent to which EF impairment contributes to reduced QoL in children with NF1, as perceived by the children themselves (self-report) or by their parents (proxy report). Based on available empirical data, we expected to find (1) self- and proxy reports of reduced QoL among the children with NF1, (2) major EF deficits, and (3) a negative impact of these deficits on QoL.

## Methods

### Participants

The clinical sample was recruited from a referral center for NF1 located in a university hospital. Participation was voluntary and offered to all parents during medical consultations carried out between May 2013 and March 2016. To be included, children had to meet the diagnostic criteria of the National Institutes of Health Consensus Conference [1] and be aged between 8 and 12 years. Learning disabilities were neither an inclusion nor an exclusion criterion, to avoid recruitment bias [13]. The exclusion criteria were epilepsy or brain tumor in the context of NF1, history of another neurological or psychiatric pathology, sensory disorder incompatible with testing (especially symptomatic optic chiasm glioma), and insufficient French language proficiency. All 52 families to whom the study was proposed agreed to participate. One child was excluded because of a prior history of head trauma, and 11 others were not included in the analyses, owing to too many missing data. The final sample therefore comprised 40 children with NF1.

Healthy control children were matched with the clinical sample for age, sex, and mean parental education level, as the standardized psychometric tests available to assess EFs in France have not all been validated with French children. We recruited a total of 71 control children through different networks (schools, holiday and leisure centers). The inclusion criteria were the same as for the clinical sample, apart from the fact that they were not expected to have NF1 or a history of learning disabilities. We used the Full Scale Intellectual Quotient (*M* = 100, *SD* = 15) derived from the four primary indices of the French Wechsler Intelligence Scale for Children-4th Edition [14] to exclude intellectual disability. A total of 12 children were not retained, owing to a high number of missing data, and three others were removed from the study because of suspected attention deficit hyperactivity disorder. The final control sample therefore consisted of 56 children.

### Assessment

General health-related QoL was measured with the validated French-language version of Kidscreen-52 [15]. Children (self-report) and their parents (proxy report) responded to 52 questions covering 10 QoL domains (Physical wellbeing, Psychological wellbeing, Moods and emotions, Self-perception, Autonomy, Parent relations and home life, Social support and peers, School environment, Social acceptance, Financial resources) on a 5-point scale. The raw scores (*M* = 50, *SD* = 10) were converted into *T* scores.

To take the current recommendations for EF assessment [16] into account, we used both direct (i.e., psychometric tests of the children’s performances in an examination setting) and indirect (behavioral inventory) measures. More specifically, we combined seven performance-based tests with two behavioral inventories targeting daily life. Three of the performance-based tests (i.e., Rey–Osterrieth complex figure (ROCF), Stroop test, and Modified Wisconsin Card Sorting Test) do not have a validated French-language version, but were selected because of their clinical sensitivity in NF1 [6, 17, 18]. For these tests, we used raw scores. Four other tests were administered, namely the two barrages test (T2B) [19], the Digit span and Letter-number sequencing subtests of the Wechsler Intelligence Scale for Children [14], and the Auditory attention and response set from the Developmental Neuropsychological Assessment [20]. We used *z* scores for T2B and standardized scores for the Developmental Neuropsychological Assessment (*M* = 10, *SD* = 3). For the Digit span and Letter-number sequencing subtests, the scores were pooled to obtain a composite working memory index (WMI; *M* = 100, *SD* = 15), in accordance with the Wechsler manual's recommendations. The French validation [21, 22] of the Behavior Rating Inventory of Executive Function (BRIEF [23]) was completed by parents and teachers, and *T* scores were used to calculate a Global Executive Composite score and two composite indices: behavioral regulation (BRI) and metacognition (MI).

Disease severity was assessed with the revised Riccardi scale [24], ranging from 1 (*Minimal*) to 4 (*Severe*). Disease visibility was assessed with the Ablon scale [25], ranging from 1 (*Mild*) to 3 (*Severe*).

The extent of school and extracurricular support received was assumed to provide an indirect measure of learning disabilities. *School support* corresponded to the educational services put in place at school (i.e., number of months the child received help such as care, school support, personalized schooling, or personalized educational success program). *Extracurricular support* corresponded to out-of-school care (i.e., number of months the child received speech or motor therapy or psychological follow-up).

### Procedure

The study was approved by an institutional review board (CPP Est III, 12 March 2013, ID-RCB no. 2012-A00787-36) and registered with the French Data Protection Authority (CNIL; EGY/VCS/AR135993). A written consent form was signed by each child and at least one parent after they had read an information note. The testing protocol was carried out by two experienced psychologists, assisted by four Master's students.

The protocol was part of a larger study of neuropsychological disorders in NF1 (3 sessions, each lasting 150 min). The children with NF1 were seen as part of their neuropsychological assessment at the hospital, while control children were seen at home. The tests were administered in a predefined and systematized order.

### Statistical analyses

All analyses were carried out with R software (R Core Team, www.r-project.org). Differences between the NF1 and control groups on raw or standardized scores (based on calibrations) were examined using two-tailed Student *t* tests with Welch's degrees of freedom, to account for the heterogeneity of variances in the population.

To control for Type 1 errors in the context of multiple comparisons, we decided to set the alpha level at 0.01. A value of *p* ≤ 0.05 therefore only indicated a trend toward significance. Effect sizes were calculated and interpreted according to Cohen's recommendations [26]: small if 0.2 ≤ *d* < 0.5; moderate if 0.5 ≤ *d* < 0.8; large if *d* ≥ 0.8.

Linear regressions were calculated to examine the determinants of low QoL domain scores (self- and proxy reports). Two kinds of predictors were considered and selected on the basis of a critical threshold of 0.10, namely (1) low EF scores, and (2) sociodemographic (age, sex, parental education level) and disease-related (sporadic vs. familial form, severity, visibility) factors. We also included the extent of school and extracurricular support (see [27, 28]), in order to contrast it with other indicators. Any missing data for a regression were imputed with the Amelia 2 package [29, 30]. The data were assumed to be multivariate normal. Amelia 2 uses the EMB algorithm (B for bootstrap [31]). A total of 100 completed datasets were created (by default) and backed up. Regression results (beta and *SE*) were pooled according to standard multiple imputation rules [32].

## Results

### Sample characteristics

The main characteristics of the clinical sample are summarized in Table 1. Children with NF1 had a mean age of 9.7 years (range: 8–12.7 years), and 35% of them had familial NF1. The distribution of severity levels (Riccardi scale) showed a predominance of minimal (40%) and moderate (37.5%) forms, followed by mild (22.5%) forms. In terms of visibility (Ablon scale), most of the children (75%) had mild forms, compared with 20% with moderate forms, and just 5% with severe forms. Mean parental education level was 13 years (range = 7–17.5 years; averaged across the two parents). Eleven children (i.e., 27.5% of the clinical sample) met DSM-5 criteria [33] for attention deficit hyperactivity disorder. A systematic exploration of the symptoms of inattention and hyperactivity/impulsivity was performed during the routine clinical interview (semi-structured interview). Conners’ rating scales [34] (French translation [35]) were used in a complementary way to examine the extent to which difficulties were found in two different life contexts, namely home and school.When the questionnaires were not returned, this aspect of the diagnosis was only based on the clinical interview with the parents [36]. Three children were receiving methylphenidate treatment at the time of their assessment, which they had to discontinue 48 h before the examination.

**Table 1:** Sociodemographic and clinical characteristics of the study samples

Sociodemographic and clinical characteristics	Children with NF1 (*n* = 40)		Healthy controls (*n* = 56)		Comparison	
	Mean or number (%)	*SD* (range)	Mean or number (%)	*SD* (range)	*t* or χ^2^	*p* Value
Age in years	9.7	1.4 (8–12.7)	9.7	1.4 (8–12.9)	*t*(94) = 0.049	0.961
Boys/girls	15/25	–	25/31	–	χ^2^ = 0.490	0.484
PEL in years	12.81	1.99 (7–17.5)	13.37	1.93 (9.5–17)	*t*(94) = 1.364	0.176
Handedness (Edinburgh)	54.45	54.91 (− 80–100)	51.96	57.63 (− 100–100)	*t*(94) = 0.212	0.832
Familial NF1	14 (35.0)	–	–	–	–	
ADHD comorbidity	11 (27.5)	–	–	–	–	
Severity, 1/2/3/4	9/16/15/0	–	–	–	–	
Visibility, 1/2/3	30/8/2	–	–	–	–	
FSIQ^a^	85.62^b^	14.05 (57–115)	102.71	12.59 (71–128)	*t*(93) = 6.209	< 0.001
School support in months	18.13	19.81 (0–80)	0.79^c^	2.35 (0–10)	*t*(91) = 6.323	< 0.001
Extracurricular support in months	33.54	29.32 (0–100)	5.49^d^	12.31 (0–60)	*t*(92) = 6.327	< 0.001

^a^Standardized score (*M* = 100, *SD* = 15)

^b^*n* = 39

^c^*n* = 53

^d^*n* = 54

PEL = parental education level; FSIQ = full scale intellectual quotient. Severity is based on Riccardi scale, ranging from 1 (minimal) to 4 (severe). Visibility is based on Ablon scale, ranging from 1 (mild) to 3 (severe)

The mean Full Scale Intelligence Quotient of children with NF1 was significantly lower than that of the control sample (*M* = 85.62 vs. 102.71, *p* < 0.001). Children with NF1 received significantly more school and extracurricular support than controls (all *p*s < 0.001).

### Quality of life

Mean scores on the different QoL domains are set out in Table 2. The Financial Resources domain was excluded from the analyses, owing to a high number of missing responses (12.2%), in contrast to the other domains (below 5%). Compared with the control group, self-reported QoL tended to be lower for two domains, namely Social support and peers, and School environment (*p* < 0.05, small effect size). Proxy-reported QoL of children with NF1 was significantly lower or tended to be lower for four of the nine domains, namely School environment, Social support and peers, and Physical wellbeing (*p* < 0.01, moderate effect sizes), and Moods and emotions (*p* < 0.05, small effect sizes). Social acceptance, Parent relationships and home life, Psychological wellbeing, Self-perception and Autonomy scores failed to reach significance.

**Table 2:** Scores on the Kidscreen-52 quality of life assessment for children with NF1 and healthy controls

Quality of life scores^a^	Children with NF1			Healthy controls			*t*(*df*)^b^	*p* Value^*b*^	Cohen’s *d*
	*n*	Mean	(*SD*)	*n*	Mean	(*SD*)			
*Physical wellbeing*									
Children	40	48.87	9.70	56	52.88	9.86	1.981 (85.01)	0.051	0.409
Parents	40	45.48	7.87	56	50.53	9.69	2.815 (92.37)	0.006	0.573
*Psychological wellbeing*									
Children	40	51.77	8.19	56	54.33	10.27	1.360 (92.79)	0.177	0.276
Parents	40	47.51	11.46	56	50.62	9.65	1.400 (74.88)	0.166	0.294
*Moods and emotions*									
Children	39	56.38	12.51	56	60.09	9.34	1.572 (66.31)	0.121	0.336
Parents	40	54.20	11.98	56	58.92	8.95	2.104 (68.57)	0.039	0.446
*Self-perception*									
Children	39	56.35	10.10	56	56.43	9.61	0.039 (79.28)	0.969	0.008
Parents	40	49.57	10.44	56	49.89	7.89	0.162 (69.13)	0.872	0.034
*Autonomy*									
Children	39	46.28	10.22	56	49.58	9.96	1.564 (80.55)	0.122	0.327
Parents	40	43.47	8.99	56	44.77	7.41	0.751 (73.74)	0.455	0.158
*Parental relations and home life*									
Children	39	47.68	9.01	56	50.58	11.13	1.400 (90.83)	0.165	0.286
Parents	40	42.84	10.86	56	46.41	9.551	1.669 (77.26)	0.099	0.349
*Social support and peers*									
Children	39	46.01	14.74	56	52.15	11.55	2.178 (68.78)	0.033	0.464
Parents	39	37.30	12.37	56	44.89	9.82	3.196 (69.45)	0.002	0.680
*School environment*									
Children	39	53.40	12.38	56	58.21	10.02	2.007 (70.44)	0.049	0.426
Parents	39	46.36	9.98	56	53.15	7.84	3.553 (68.90)	< 0.001	0.756
*Social acceptance*									
Children	39	49.52	11.97	56	52.02	7.65	1.149 (59.34)	0.255	0.249
Parents	40	46.84	10.99	56	50.76	8.73	1.875 (71.73)	0.065	0.395

^a^Standardized *T* score (*M* = 50, *SD* = 10); lower scores indicate poorer quality of life

^b^All values shown correspond to the degrees of freedom, *t* and *p* corrected (Welch correction)

### Executive functions

Descriptive statistics (mean scores and standard deviations) for performance-based EF tests and BRIEF for the children with NF1 and healthy controls are summarized in Table 3. Scores differed significantly between groups for six out of nine performance-based measures (all *p*s < 0.01): WMI, ROCF, and Auditory attention and response set (large effect sizes), Stroop time interference and uncorrected errors, and T2B accuracy (moderate-to-large effect sizes). The difference for T2B speed tended toward significance (*p* < 0.05; small effect size). The two groups had comparable scores on the Modified Wisconsin Card Sorting Test. Parents’ and teachers’ BRIEF ratings (see Table 3) were significantly higher for the children with NF1, in terms of the BRI, MI, and Global Executive Composite score (all *p*s < 0.001; large effect sizes).

**Table 3:** Results of executive function assessment for children with NF1 and healthy controls

Executive function assessment	Children with NF1			Healthy controls			*t*(*df*)^e^	*p* value^e^	Cohen’s *d*
	*n*	Mean	(*SD*)	*n*	Mean	(*SD*)			
*Performance-based tests*									
ROCF, planning index^a^	39	4.49	4.11	56	1.20	3.11	4.231 (66.96)	< 0.001	0.904
Stroop, time interference^a^	40	129.40	57.35	56	95.75	45.81	3.076 (72.04)	0.003	0.648
Stroop, errors^a^	40	4.28	4.92	56	1.41	2.02	3.483 (48.44)	0.001	0.763
WMI^b^	39	83.23	12.98	56	100.48	14.42	6.086 (87.01)	< 0.001	1.257
MCST, categories^a^	40	3.95	1.52	56	4.11	1.34	0.524 (77.63)	0.602	0.110
MCST, perseverations^a^	40	4.40	3.71	56	4.05	3.00	0.488 (72.74)	0.627	0.103
T2B, speed^a^	38	104.50	32.26	55	119.80	32.53	2.241 (80.15)	0.028	0.472
T2B, accuracy^a^	38	8.16	6.40	55	4.23	4.03	3.351 (57.17)	0.001	0.734
Auditory attention^c^	35	8.86	2.10	55	10.46	1.18	4.101 (47.87)	< 0.001	0.936
*Behavioral inventories*									
BRIEF-parent, GEC^d^	40	63.35	15.05	53	46.75	9.81	6.068 (63.13)	< 0.001	1.306
BRIEF-parent, BRI_d_	40	61.45	16.76	53	47.30	9.69	4.770 (58.38)	< 0.001	1.033
BRIEF-parent, MI_d_	40	62.80	13.11	53	46.85	9.31	6.548 (67.07)	< 0.001	1.403
BRIEF-teacher, GEC_d_	34	64.29	13.30	45	47.89	14.02	5.303 (73.07)	< 0.001	1.201
BRIEF-teacher, BRI_d_	34	61.21	17.31	45	46.04	6.34	4.866 (39.73)	< 0.001	1.163
BRIEF-teacher, MI_d_	34	65.21	13.03	45	46.93	7.34	7.344 (48.64)	< 0.001	1.728

ROCF = Rey–Osterrieth complex figure; WMI = working memory index; MCST = modified card sorting test; T2B = two barrages test; BRIEF = behavior rating inventory of executive function; GEC = global executive composite; BRI = behavioral regulation index; MI = metacognition index

^a^Raw score

^b^Standardized score (*M* = 100, *SD* = 15)

^c^Standardized scale score (*M* = 10, *SD* = 3)

^d^Standardized *T* score (*M* = 50, *SD* = 10)

^e^All values shown correspond to the degrees of freedom, *t* and *p* corrected (Welch correction)

### Predicting quality of life

The results of the regression analysis to assess potential predictors of reduced QoL (*p* = 0.10 threshold), distinguishing between EFs and sociodemographic or disease-related factors, are detailed in Table 4. For EFs, we generally expected to observe negative relationships, given that for the majority of executive measures, higher scores are associated with more severe difficulties, whereas lower QoL scores are associated with poorer QoL. For WMI and the Auditory attention and response set, we expected to observe positive relationships, as higher scores are associated with better performances.

**Table 4:** Predictors of quality of life in children with NF1 (*n* = 40). Results of regression analysis

				QoL					
	Child self-report			Parent proxy report					
	Ph WB	Soc sup	Sch env	Ph WB	Moods em	Par rel	Soc sup	Sch env	Soc Acc
Model 1 *Executive functions*									
ROCF, planning index		− 1.7 (0.7)**	− 1.2 (0.6)*						− 1.3 (0.4)***
Stroop, errors									
WMI		− 0.4 (0.2)*					− 0.4 (0.2)*		
T2B speed			− 0.2 (0.1)*			− 0.1 (0.1)*		− 0.1 (0.1)*	− 0.1 (0.1)*
T2B accuracy									
Auditory attention									
BRIEF parents, BRI					− 0.5 (0.2)*				− 0.4 (0.2)*
BRIEF parents, MI							− 0.6 (0.3)^†^	− 0.5 (0.2)**	
BRIEF teacher, BRI									
BRIEF teacher, MI		− 0.4 (0.2)^†^	− 0.4 (0.2)^†^	− 0.3 (0.1)*					− 0.5 (0.2)**
Model 2 *Sociodemographic and disease-related factors*									
Age						− 0.2 (0.1)*			
Sex (boys)	2.7 (1.5)^†^		9.5 (4.2)*		10.6 (3.7)**				7.2 (3.6)*
PEL									
Sporadic NF1									
Severity					− 4.7 (2.8)^†^	− 6.0 (2.8)*	− 8.3 (3.1)**		
Visibility	− 4.0 (1.7)*	− 15.9 (5.7)**	− 9.6 (4.9)*						
Indirect LD indicators									
School support	− 0.27 (0.1)^†^								
Extracurricular support									− 0.1 (0.1)^†^

QoL = quality of life; PhWB = physical wellbeing; Soc sup = social support and peers; Sch env = school environment; Moods em = moods and emotions; Par rel = parental relations and home life; Soc acc = social acceptance; ROCF = Rey–Osterrieth complex figure; WMI = working memory index; T2B = two barrages test; BRIEF = behavior rating inventory of executive function; GEC = global executive composite; BRI = behavioral regulation index; MI = metacognition index; PEL = parental education level; LD = learning disabilities. Severity is based on Riccardi scale. Visibility is based on Ablon scale

***p < 0.001; **p < 0.01; *p < 0.05; ^†^*p* < 0.10

Analysis of EFs in the first model showed that the difficulties observed on two performance-based tasks (ROCF and T2B), and reported in the BRIEF teacher form (MI), contributed negatively to self-reported QoL for two of the three weakened domains, namely Social support and peers, and School environment. Contrary to expectations, the relationships between WMI and these two domains were negative. Analysis of the results for proxy-reported QoL indicated that ROCF and T2B scores were negatively related to three domains (i.e. Social acceptance, Parent relations and home life, and School environment). Once again, there was an unexpected negative relationship between WMI and the Social support and peers domain. In addition, most of the proxy-reported QoL domains were significantly and negatively related to BRIEF parent form scores for BRI (Moods and emotions and Social acceptance) and MI (Social support and peers, School environment), and to BRIEF teacher form scores for MI (Physical wellbeing, Social acceptance).

Regarding sociodemographic and disease-related factors, analysis showed that children’s age was negatively associated with proxy-reported QoL for the Parent relationships and home life domain. In addition, a positive relationship was identified between male sex and self-reported QoL for the Physical wellbeing and School environment domains. The same relationship was observed with proxy-reported QoL for the Moods and emotions and Social acceptance domains. Disease severity was negatively related to proxy-reported QoL for the Moods and emotions, Parent relations and home life, and Social support and peers domains, while a negative relationship was found between disease visibility and self-reported QoL for all the weakened domains (Physical wellbeing, Social support and peers, and School environment). Indicators of learning disabilities (school or extracurricular support) tended to negatively contribute to self-reported QoL for Physical wellbeing, and to proxy-reported QoL for Social acceptance. Finally, neither parental education level nor familial versus sporadic form of the disease significantly influenced QoL scores.

## Discussion

This prospective study was designed to examine the potential impact of EF deficits on general health-related QoL, as perceived by children with NF1 and their parents.

In accordance with our first hypothesis, we found a significant decrease in QoL among children with NF1 in several domains. Two QoL domains, namely School environment and Social support and peers, were perceived as weakened by both the children (only trends after control for Type 1 errors) and their parents (largest effect sizes). This reflected school’s lack of appeal and negative feelings toward the school context, as well as a sense of social exclusion and difficulty establishing friendships. The reduced QoL in our clinical sample confirmed the results of previous studies [27, 28, 37–39]. It appeared to be more pronounced for parents (four domains concerned; moderate effect sizes for three of them) than for their children (only two domains concerned; small effect sizes). These results are reminiscent of previous findings whereby the impact of NF1 on QoL is primarily or preferentially perceived by parents [27, 28], although children also express a degree of illbeing [37, 38].

In accordance with our second hypothesis, results also confirmed that EFs are particularly vulnerable in children with NF1 [5, 6]. The latter scored significantly lower than healthy controls on most performance-based tests (i.e., six of the nine measures we selected), with generally moderate-to-large effect sizes. The appraisal of EFs in daily life (BRIEF) confirmed massive impairments affecting both behavioral regulation (BRI) and metacognition (MI), according to both parents and teachers (large size effects).

The exploratory regression analysis confirmed our third hypothesis that EF impairments are significant predictors of reduced QoL in children with NF1. This relationship was found for at least one of the EF measures in eight of the nine QoL domains we examined. The convergence of results from performance-based tests and questionnaires indicated that three QoL domains were particularly impacted by EF deficits, namely Social support and peers, School environment, and Social acceptance (proxy reports only for the latter). Moreover, our data showed that both performance-based tests and parent and/or teacher observations were complementary indicators of children’s wellbeing. These results further illustrate the essential role of EFs in psychological development in the broadest sense, including behavioral regulation, social knowledge integration, and academic achievement [9]. This is corroborated by recent studies showing that executive dysfunction in children with NF1 helps to explain both learning disabilities [40] and poor adaptive behavior [41]. It should, however, be noted that our results contrasted with those of another recent study carried out in a large group of children with NF1 [42], which failed to find a significant link between EF difficulties and reduced QoL. However, the tests used were not the same as in our study, and did not include measures of EFs in daily living, which may help to explain the divergent results.

Furthermore, the unexpected direction of the relationship between WMI and the Social support and peers QoL domain contrasted with the other relationships identified in our data. We have no obvious explanation for this result. It is possible that this composite measure of working memory, which is derived from subtests of the Wechsler intelligence scales, interacts differently with certain domains of perceived QoL, compared with other EF measures. Perhaps, for example, better general cognitive skills combined with working memory resources contribute to greater awareness of difficulties, thus resulting in poorer QoL. However, this interpretation is still very speculative, and further studies are needed to better understand this result.

Finally, regarding sociodemographic and disease-related factors, children’s age only partially influenced QoL, in accordance with several previous studies [27, 38, 43]. Male sex appeared to be associated with better QoL (self- and proxy reports), which may be explained by higher societal expectations for girls, although there are contrasting results in the literature [27, 38]. The nonpredictive nature of parental education level was consistent with some previous findings [38], but again there is no consensus [27, 39]. No positive or negative impact on QoL of familial forms of the disease [3] was found in our sample. By contrast, parents perceived a negative effect of disease severity on QoL (for similar results, see [38]), while the impact of visibility was negatively perceived by children (for similar results, see [27]), suggesting that children and parents are not sensitive to the same markers of the disease. The impact of academic difficulties on QoL was perceived by both children and their parents to be limited (see also [27]), reflecting a possible lack of sensitivity of these indirect indicators of school failure, in contrast to EF tests.

The present study had several potential limitations. First, the small sample size means that the results should be interpreted with caution, especially given the inherent variability of this disease. Another study with a larger sample is required, as well as a comparison with other rare diseases. Second, as highlighted by other studies, it would have been preferable to use a QoL questionnaire specially developed for NF1, especially as this would have helped to reduce missing data [44–46]. Such a questionnaire was recently developed [47], but was not available for use at the time of the present study. It is also essential to differentiate between the QoL judgments of fathers versus mothers, in order to highlight any judgment bias, which was not possible here. In addition, phenotype variability, particularly at the neuropsychological level, may contribute to group effects, hence the need for studies of clinical profiles, for example in cluster form [41].

## Conclusions

This exploratory study confirms that EF deficits have a significant impact on the QoL of children with NF1, as perceived by both the children themselves and their parents, regardless of the indirect indicators of learning disabilities. Social interactions and School environment are the most vulnerable QoL domains, linked to the fundamental role of EFs in psychological development. Our results also show the importance of combining performance-based tests with parents’ and teachers’ observations in everyday life, to ensure a comprehensive approach to EF deficits and their impact on QoL. Finally, this research encourages more systematic implementation of interventions to manage EF difficulties, in order to improve the wellbeing of children with NF1.

## Data Availability

Raw data are available on request.

## Abbreviations

NF1: Neurofibromatosis type 1
QoL: Quality of life
EFs: Executive functions
ROCF: Rey–Osterrieth complex figure
T2B: Two barrages test
WMI: Working memory index
BRIEF: Behavior rating inventory of executive function
BRI: Behavioral regulation index
MI: Metacognition index
